# Redox Dysregulation in the Abdominal Aortic Aneurysm Wall: Ferroptosis and Experimental Therapeutic Opportunities

**DOI:** 10.3390/jcdd13100510

**Published:** 2026-10-10

**Authors:** Yi Huang, Feng Zhu, Chuanqi Cai, Dan Shang

**Affiliations:** 1 Department of Vascular Surgery, Union Hospital, Tongji Medical College, Huazhong University of Science and Technology, Wuhan 430022, China; 2 Department of Vascular Surgery, Hubei Provincial Hospital of Traditional Chinese Medicine, Wuhan 430061, China

**Keywords:** abdominal aortic aneurysm, redox dysregulation, ferroptosis, iron metabolism, lipid peroxidation, antioxidant defence

## Abstract

Abdominal aortic aneurysm (AAA) is a spatially heterogeneous disease of the aortic wall for which no pharmacological treatment has reliably arrested enlargement. The term oxidative stress encompasses processes that differ in chemical form, anatomical location and cellular effect. This narrative review examines haem and redox-active iron, phospholipid peroxidation and antioxidant defence at the intraluminal thrombus–wall interface and within medial and inflammatory cells. We distinguish ferroptosis from redox effects on coagulation, migration and secretion and relate these functions to wall remodelling. Experimental studies show that manipulating iron uptake and ferritin turnover, membrane phospholipid susceptibility or peroxide detoxification can alter vascular smooth muscle cell survival, macrophage behaviour and aortic injury. The cellular function affected helps determine which process an intervention should preserve or suppress. Based on evidence from human tissue and animal models, we review opportunities to localise, manipulate and monitor these processes and assess their relevance to established AAA. Testing after confirmation of aneurysm formation, with target engagement in defined wall cells and parallel assessment of systemic effects, is needed to determine whether these approaches can limit continued enlargement.

## 1. Introduction

Abdominal aortic aneurysm (AAA) is a progressive disease in which medial cell loss, extracellular matrix degradation, inflammation and intraluminal thrombosis weaken the aortic wall. Enlargement is usually clinically silent, whereas rupture carries a high risk of death. Surveillance and cardiovascular risk reduction remain the main strategies for small asymptomatic AAAs. Repair is recommended when size, growth or symptoms indicate an unacceptable rupture risk. No pharmacological treatment has reliably arrested enlargement or removed the subsequent need for repair [1,2,3,4]. This therapeutic gap directs attention to the biological processes that remain active within the aneurysmal wall and sustain its remodelling after the lesion has formed.

Redox injury develops within a heterogeneous wall, where local exposure, cellular composition and antioxidant capacity vary. Grouping these processes under oxidative stress can obscure which chemical species act on which cells and what functions they alter.

Ferroptosis research has linked iron availability, phospholipid peroxidation and failed antioxidant defence to cell death [5,6,7,8]. Genetic and pharmacological studies in vascular and aortic disease have examined how iron uptake and storage, membrane lipid susceptibility and peroxide detoxification affect cellular and aortic outcomes [9,10]. Lipid peroxidation can also alter coagulation, migration and secretion in surviving cells. The distinction matters therapeutically: preserving a structural cell and suppressing an inflammatory cell’s damaging activity are different aims, even when both interventions lower oxidative markers.

This narrative review draws on PubMed and Web of Science searches through July 2026, initiated with the terms ‘ferroptosis’ and ‘aneurysm’ and expanded through topic-specific searches. Additional searches during revision retained the same publication cutoff. Human and experimental AAA studies inform the disease-specific discussion, with non-AAA studies providing context for the underlying pathways.

This review examines where ferroptosis contributes to AAA wall injury and how the affected cell types and timing of intervention shape the prospects for treating established AAA.

## 2. Iron Handling, Lipid Peroxidation and Antioxidant Defence in the Aneurysmal Wall

The architecture of the aneurysmal wall determines where redox processes can arise and which cells they can affect. Medial vascular smooth muscle cells (VSMCs) are depleted or phenotypically altered, the extracellular matrix is fragmented, and immune cells accumulate in the media and adventitia [11]. In many established AAAs, an intraluminal thrombus (ILT) covers the luminal surface and contains fibrin, platelets, leukocytes, cellular debris and erythrocytes [12,13,14]. Its cellular content and permeability vary across layers, while the underlying wall differs in cellular composition and structural integrity [15,16]. These features place blood-derived material, inflammatory activity and vulnerable structural cells in adjacent but distinct compartments.

The ILT–wall interface does not impose a uniform redox environment. A thick ILT has been associated with local wall hypoxia and reduced wall strength [17], whereas another study found greater oxidative stress and proteolytic-enzyme expression beneath thin rather than thick ILTs [18]. These different endpoints do not support a simple increase in oxidative injury with increasing thrombus thickness. Tunica-specific transcriptomics and regional matrix metalloproteinase measurements also reveal differences between medial and adventitial programmes and between the wall and ILT [19,20]. Bulk tissue measurements consequently combine signals from regions differing in blood product exposure, oxygenation and cell composition.

Blood products provide several chemically distinct inputs to this environment. Human ILT contains relatively intact erythrocytes towards the lumen and greater haemoglobin degradation in deeper layers [21]. Ferryl haemoglobin has also been localised extracellularly and in neutrophils and macrophages within haemorrhagic AAA walls [22]. In primary human neutrophil experiments, ferryl haemoglobin stimulated myeloperoxidase and elastase release; conditioned medium from exposed neutrophils fragmented elastin in human aortic sections [22]. This whole-medium experiment did not distinguish secreted enzyme effects from residual ferryl haemoglobin. Haemoglobin exposure and conditioned medium from AAA adventitia promoted monocyte recruitment and a CD163-high macrophage phenotype with increased haemoglobin uptake in human monocyte cultures [23]. These responses place inflammatory activation, recruitment and haemoglobin disposal alongside one another as potential consequences of blood-derived exposure.

Stored ferric iron, haem-bound iron and the intracellular labile pool differ in their availability for lipid oxidation [24,25]. Human AAA studies have detected iron deposits in the ILT and wall, ferritin in infiltrating leukocytes and morphologically smooth muscle-like cells, and increased wall iron associated with oxidative DNA damage and macrophage infiltration [21,26]. Histochemistry has localised peroxidase activity to diseased intimal and residual medial regions, with haemosiderin deposits in areas adjacent to the ILT [27]. ILT and adventitial products also induced iron-sensitive oxidative responses in cultured aortic VSMCs [27]. Whether haem-derived iron from human ILT reaches the labile pool of a defined wall cell and causes ferroptotic death remains unresolved.

Lipid oxidation affects both membranes and extracellular lipoproteins. Human aneurysmal aorta shows increased superoxide, NADPH oxidase activity and nitrotyrosine, together with lipid hydroperoxides, conjugated dienes and adducts formed from malondialdehyde [28,29,30,31,32]. Lipoprotein function is also altered within the lesion and circulation. Modified high-density lipoprotein (HDL) elicits a specific antibody response [33]. In aortic tissue, *OLR1* mRNA, encoding lectin-like oxidised low-density lipoprotein receptor 1 (LOX-1), showed only a nonsignificant upward trend in AAA compared with aortic occlusive disease [34]. Oxidation of apolipoprotein A1 weakens the antioxidant capacity of HDL [27,35].

Phospholipid hydroperoxides reflect primary membrane oxidation, whereas malondialdehyde and related aldehydes arise during decomposition and can form protein adducts. Oxidised lipoproteins introduce extracellular substrates and signalling effects. Membrane susceptibility, peroxide accumulation and downstream damage consequently need not change in parallel when an intervention alters phospholipid composition or peroxide removal.

Antioxidant responses can coexist with injury and may reveal adaptation within an exposed wall region. Activities of superoxide dismutase, catalase, glutathione peroxidase and glutathione reductase differ beneath thin and thick ILTs, yet higher activity does not necessarily normalise local oxidative stress [18]. Haem oxygenase 1 (HO-1) protein is increased in human AAA; within elective AAA specimens, greater expression was associated with lower H_2_O_2_, thiobarbituric acid-reactive substances and protein-carbonyl measurements [36]. HO-1 degrades haem, but protein abundance does not establish the rate of haem turnover or the fate of the released iron. Increased HO-1 therefore cannot by itself demonstrate an expanded labile iron pool. Its functional meaning depends on whether haem disposal is coordinated with iron sequestration or export and on the oxidant burden that remains.

**Figure 1:**
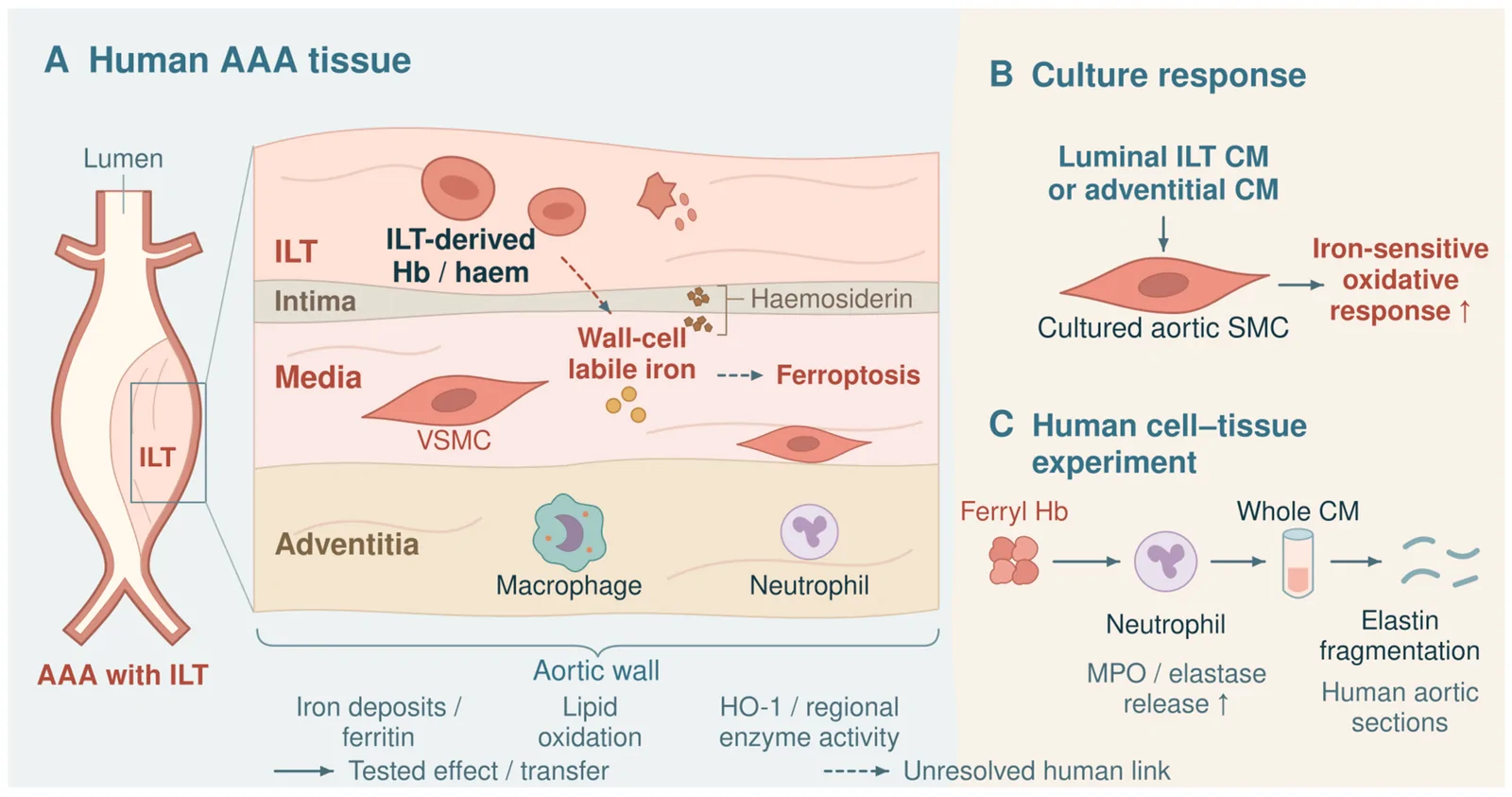
Redox environments and tissue–cell interactions in abdominal aortic aneurysm (AAA). (**A**) Human tissue observations at the intraluminal thrombus (ILT)–wall interface. (**B**) Tissue-to-cell conditioned-medium (CM) experiments using the luminal ILT or adventitia, tested separately. (**C**) Neutrophil-to-aortic-tissue CM transfer following ferryl haemoglobin exposure. These panels distinguish the anatomical context from experimentally tested interactions. Solid arrows indicate tested effects or transfer; dashed arrows identify unresolved links between ILT-derived haem and ferroptosis in human wall cells; unheaded lines indicate anatomical locations. Brown aggregates represent tissue haemosiderin deposits, whereas gold circles denote the proposed labile iron pool. Peach, teal and purple cells represent vascular smooth muscle cells (VSMCs), macrophages and neutrophils, respectively. Anatomy and node positions are schematic. Upward arrows indicate increases relative to the experimental comparison. Hb, haemoglobin; HO-1, haem oxygenase-1; MPO, myeloperoxidase; SMC, smooth muscle cell.

Human AAA consequently provides a spatial map of blood-derived exposure, iron retention, lipid modification and adaptive defence (Figure 1). Within this environment, the biological consequence depends on how wall cells handle iron, remodel lipids and remove peroxides. Experimental perturbations can identify which of these operations changes cell survival, recruitment or matrix injury.

## 3. Experimental Manipulation of Redox Pathways and Ferroptosis in AAA

The cellular consequences of redox stress extend from altered migration, coagulation and secretion to lethal membrane injury. Here, ferroptosis denotes cell death accompanied by lipid peroxidation and supported by functional evidence identifying iron-dependent lethal lipid oxidation [5,37]. Changes in iron, reactive oxygen species (ROS), malondialdehyde, glutathione or glutathione peroxidase 4 (GPX4), even with responses to radical-trapping antioxidants, do not establish ferroptosis without a death endpoint. Reduced aneurysm burden alone does not establish that protection is mediated by preventing ferroptotic cell death.

### 3.1. Iron Access, Storage and Turnover

Receptor-mediated uptake links extracellular iron supply to VSMC injury. Ganglioside GM3 and its synthase ST3GAL5 were reduced in VSMCs from human AAA specimens and angiotensin II (Ang II)-treated mice. GM3 bound the extracellular domain of transferrin receptor 1 (TFR1) and impaired holo-transferrin binding. In cultured human aortic VSMCs, silencing *ST3GAL5* increased the calcein-assessed labile iron pool, lipid peroxidation and propidium iodide (PI)-positive cell death. Ferrostatin-1 attenuated death, while knockdown of *TFRC* (encoding TFR1) rescued the iron, oxidation and death responses, connecting the uptake mechanism to ferroptosis in this cellular setting [38]. Smooth muscle-specific *St3gal5* deletion aggravated experimental AAA; GM3 supplementation from the start of Ang II infusion reduced aneurysm formation.

Iron already stored in ferritin can become available through lysosomal turnover. Mechanistic studies outside AAA identify ferritinophagy mediated by nuclear receptor coactivator 4 (NCOA4), a ferritin cargo receptor, as one route through which stored iron increases ferroptosis susceptibility [39,40]. Proprotein convertase subtilisin/kexin type 9 (PCSK9) was enriched in VSMCs from human AAA tissue and experimental models. Manipulating PCSK9 altered cellular iron, lipid peroxidation indices, antioxidant measures and metabolic viability; ferritin–lysosome colocalisation and bafilomycin A1 rescue supported lysosomal ferritin turnover [41]. Smooth muscle-targeted PCSK9 overexpression worsened aortic dilatation and matrix injury, whereas the PCSK9-degrading peptide Cadd4 attenuated these outcomes. Separate studies linked the RNA methylation regulator WT1-associated protein (WTAP) to Ang II responses in cultured human aortic VSMCs through its regulation of *PCSK9* or brain abundant membrane attached signal protein 1 (*BASP1*) transcripts [42,43]. These upstream regulators extend the routes through which Ang II exposure can modify the VSMC injury response.

Iron export extends this discussion to cellular maintenance and inflammation. Hepcidin limits ferroportin-mediated export. Smooth muscle-specific deletion of *Hamp*, or expression of hepcidin-resistant ferroportin, reduced iron retention and aggravated Ang II-induced aortic disease [44]. Loss of smooth muscle hepcidin increased lipocalin 2 (LCN2), impaired autophagy and promoted neutrophil infiltration; LCN2 neutralisation restored autophagy and attenuated the heightened injury. Iron retention in this setting therefore supports wall maintenance through autophagy and control of inflammatory recruitment.

Restricting receptor-mediated uptake reduced lethal lipid oxidation, whereas preserving hepcidin-dependent retention supported autophagy and limited inflammation. Although obtained in separate experimental settings, these different requirements for iron make the affected process central to intervention: lowering cellular iron may reduce one route of injury while impairing another route of wall maintenance.

### 3.2. Phospholipid Remodelling and Cellular Outcomes

Accessible iron does not determine tissue injury on its own; oxidisable membrane substrates and their biological function also matter. Enzymatically oxidised phospholipids were detected in human AAA wall and ILT, while global deletion of the lipoxygenase genes *Alox12* or *Alox15* and inhibition of factor Xa reduced AAA development in Ang II-infused mice [45]. These experiments implicate coagulation in the effects of oxidised phospholipids. Their distribution also mattered: intravenous procoagulant lipid membranes given during induction reduced aneurysm formation, while an acute dosing experiment produced a bleeding phenotype. This was consistent with systemic clotting-factor activation and consumption limiting availability at the lesion, although factor localisation during chronic dosing was not resolved. A lower aneurysm burden can therefore coexist with a haemostatic cost, rather than necessarily indicating preserved wall cell survival.

Within VSMCs, phospholipid composition determines susceptibility to peroxidative injury. Acyl-CoA synthetase long-chain family member 4 (ACSL4) activates polyunsaturated fatty acids for incorporation into phospholipids and is a major determinant of ferroptosis sensitivity [46,47]. A study linking periodontal *Fusobacterium nucleatum* to AAA identified altered circulating isoleucine and increased VSMC H3K9 acetylation and ACSL4 expression in human disease, then examined this association experimentally [48]. Bacterial supernatant and isoleucine increased lipid oxidation and PI-positive death in cultured human aortic VSMCs; ferrostatin-1 reduced death whereas the tested caspase and necroptosis inhibitors did not. *ACSL4* knockdown suppressed lipid oxidation, and iron chelation altered the associated protein response. In mice, bacterial *ilvE* deletion or dietary isoleucine restriction began before Ang II reduced aneurysm formation and elastin injury. These findings extend the determinants of VSMC lipid susceptibility beyond intrinsic metabolism to microbial and dietary inputs.

Lipid oxidation can instead support recruitment of surviving inflammatory cells. Extracellular vesicles (EVs) from T cells with activated pyruvate kinase M2 (PKM2) increased macrophage iron, C11-BODIPY oxidation and migration, whereas vesicles from PKM2-deficient T cells produced weaker responses [49]. Ferrostatin-1, liproxstatin-1 and deferoxamine suppressed migration; direct survival measurements showed no appreciable increase in death under the tested exposure conditions. Patient-derived EVs reproduced the iron, oxidation and migration responses in a human monocytic cell line, adding functional evidence from patient material. T-cell-specific deletion of the PKM2-specific exon of *Pkm* and inhibition of EV release also reduced macrophage accumulation, matrix metalloproteinase expression, elastin disruption and aneurysm formation in the elastase model. This response provides a route by which lipid oxidation can amplify macrophage recruitment without a measurable increase in death.

The therapeutic goal therefore changes with the cellular function: preserve VSMCs, reduce recruitment of viable macrophages, or modulate procoagulant lipid activity while retaining haemostatic balance.

### 3.3. Peroxide Detoxification and Compartmentalised Antioxidant Defence

Because lipid peroxide accumulation reflects both production and removal, experiments on antioxidant defence test the other side of the balance. GPX4 reduces phospholipid hydroperoxides using glutathione, while solute carrier family 7 member 11 (SLC7A11) supplies cystine for glutathione synthesis [5,6,7]. In Ang II-exposed VSMCs, silencing *Slc7a11* or *Gpx4* weakened protection by ferrostatin-1, and the GPX4 inhibitor RSL3 produced an opposing response [50]. Ferrostatin-1 also improved metabolic viability in cultured mouse VSMCs and attenuated aortic ring injury [51]. Global GPX4 overexpression reduced lipid oxidation in the elastase–β-aminopropionitrile (BAPN) model and limited aneurysm formation and inflammation in elastase–BAPN and Ang II models [52]. In a mouse aortic VSMC line (MOVAS), GPX4 augmentation also suppressed interleukin-6 (IL-6)-induced Janus kinase 1 (JAK1)/signal transducer and activator of transcription 3 (STAT3) signalling. Together, these experiments connect phospholipid peroxide control to inflammatory signalling as well as aneurysm susceptibility.

Inflammatory signals can interrupt this defence within specific cellular compartments. Neutrophil extracellular trap (NET)-stimulated human aortic VSMCs showed glutathione depletion, increased iron and oxidation, and reduced metabolic viability; DNase I, ferrostatin-1 and phosphoinositide 3-kinase (PI3K) activation attenuated these responses [53]. Mesenchymal stromal cell (MSC)-derived vesicles acted upstream by reducing NET release, rather than directly protecting VSMCs already exposed to NETs. In a separate study, NETs altered the stability and dimerisation of the mitochondrial 2-oxoglutarate/malate carrier SLC25A11. These changes were linked to mitochondrial glutathione depletion, mitochondrial lipid oxidation and PI-positive death; SLC25A11 overexpression attenuated the death response [54]. NET exposure thus identifies two sites for protection: limiting the inflammatory input released by neutrophils and maintaining mitochondrial antioxidant capacity within VSMCs.

GPX4-centred experiments leave the redundancy of lipid-peroxide defence in AAA largely unresolved. Ferroptosis suppressor protein 1 (FSP1) regenerates ubiquinol at extramitochondrial membranes, GTP cyclohydrolase 1 (GCH1)-derived tetrahydrobiopterin (BH4) limits lipid oxidation, and a mitochondrial defence involving dihydroorotate dehydrogenase (DHODH) and coenzyme Q (CoQ) has been reported in non-AAA systems [55,56,57]. Their activity cannot be inferred from GPX4 or glutathione abundance. Aortic DHODH staining increased after global GPX4 augmentation [52], but direct functional validation of FSP1–CoQ or DHODH–CoQ ferroptosis defence was not identified in the AAA studies examined here. GCH1/BH4 has disease-specific evidence for a different function: *Gch1* deletion using Tie2-Cre increased endothelial nitric oxide synthase (eNOS) uncoupling, aortic H_2_O_2_ production and Ang II-induced aneurysm formation [58]. The model also affects myeloid cells, but it links impaired BH4-dependent redox control to aneurysm susceptibility without demonstrating ferroptosis protection. Testing the distribution and compensation of these defences across wall cells would resolve a mechanistic gap in GPX4-centred intervention.

Peroxide control also restrains inflammatory and structural injury. Menaquinone-4, Krüppel-like factor 5 (KLF5) modulation and salvianolic acid B have been linked to antioxidant responses and reduced experimental dilatation [59,60,61]. Global loss of *Prdx2*, which encodes the peroxide-detoxifying enzyme peroxiredoxin 2 (PRDX2), increased oxidative damage, inflammation, elastin degradation and dilatation [62], establishing a protective redox function; later VSMC experiments examined lipid oxidation and antioxidant responses [63]. High-mobility group box 2 (HMGB2), STAT3/HMGB2 and the transcription factor FOSB likewise affect iron, oxidative markers and inflammatory or cellular responses [64,65,66]. These findings place antioxidant defence within the control of inflammatory signalling and matrix injury, without identifying ferroptotic death as a common mediator.

Delivery systems and microbial interventions can modify several injury pathways at once [67,68,69,70]. Vesicles carrying mRNA encoding phosphatidylethanolamine-binding protein 1 (PEBP1), for example, increased its expression and reduced ROS and apoptosis in primary mouse aortic VSMCs [69]. PEBP1 can also form complexes with 15-lipoxygenases that generate pro-ferroptotic phospholipid hydroperoxides in non-AAA systems [71]. The molecular interactions underlying the protective response in the AAA study remain unresolved.

### 3.4. Macrophage Redox Responses and Paracrine Wall Injury

Macrophages connect lipid oxidation to wall injury through recruitment and paracrine signalling as well as possible lethal injury. Resolvin D1 (RvD1), acting through formyl peptide receptor 2 (FPR2), reduced NADPH oxidase 2 (NOX2)-associated oxidant production, lipid peroxidation and high-mobility group box 1 (HMGB1) release from elastase-exposed macrophages; conditioned medium from these cells induced less matrix metalloproteinase-2 (MMP-2) activity in VSMCs [72]. Global FPR2 deficiency abolished cellular effects and weakened protection against experimental dilatation.

Macrophage inflammatory output also responds to changes in intracellular antioxidant support. Small ubiquitin-like modifier (SUMO)-specific peptidase 3 (SENP3) de-SUMOylated the hydrogen sulphide-producing enzyme cystathionine γ-lyase (CTH) and accelerated its proteasomal degradation, lowering hydrogen sulphide availability [73]. In Ang II-exposed mouse bone marrow-derived macrophages, SENP3 deficiency preserved metabolic viability and limited iron accumulation and oxidative injury. Silencing *Cth* weakened antioxidant protein responses and restored pro-inflammatory transcription, while CTH inhibition with propargylglycine weakened the metabolic and redox benefit. Myeloid *Senp3* deletion reduced aneurysm severity in male and female mice in Ang II and CaCl_2_ models, with CTH inhibition counteracting the benefit in Ang II mice. These myeloid-targeted experiments link CTH-dependent redox control to lower inflammatory output and reduced aortic injury. Increased SENP3 in macrophage-rich human AAA regions places this regulator within a relevant wall population.

Liproxstatin-1 similarly reduced macrophage lipid peroxidation and HMGB1 and matrix metalloproteinase-9 output; whole conditioned medium from treated macrophages induced less MMP-2 activity and lactate dehydrogenase (LDH) release in recipient VSMCs [74]. Because liproxstatin-1 was present during conditioning, residual drug may also have contributed to the recipient-cell response. Donor-cell FPR2 dependence in RvD1 experiments [72] more specifically supports a macrophage-mediated component of protection, although individual secreted factors were not established as mediators.

**Figure 2:**
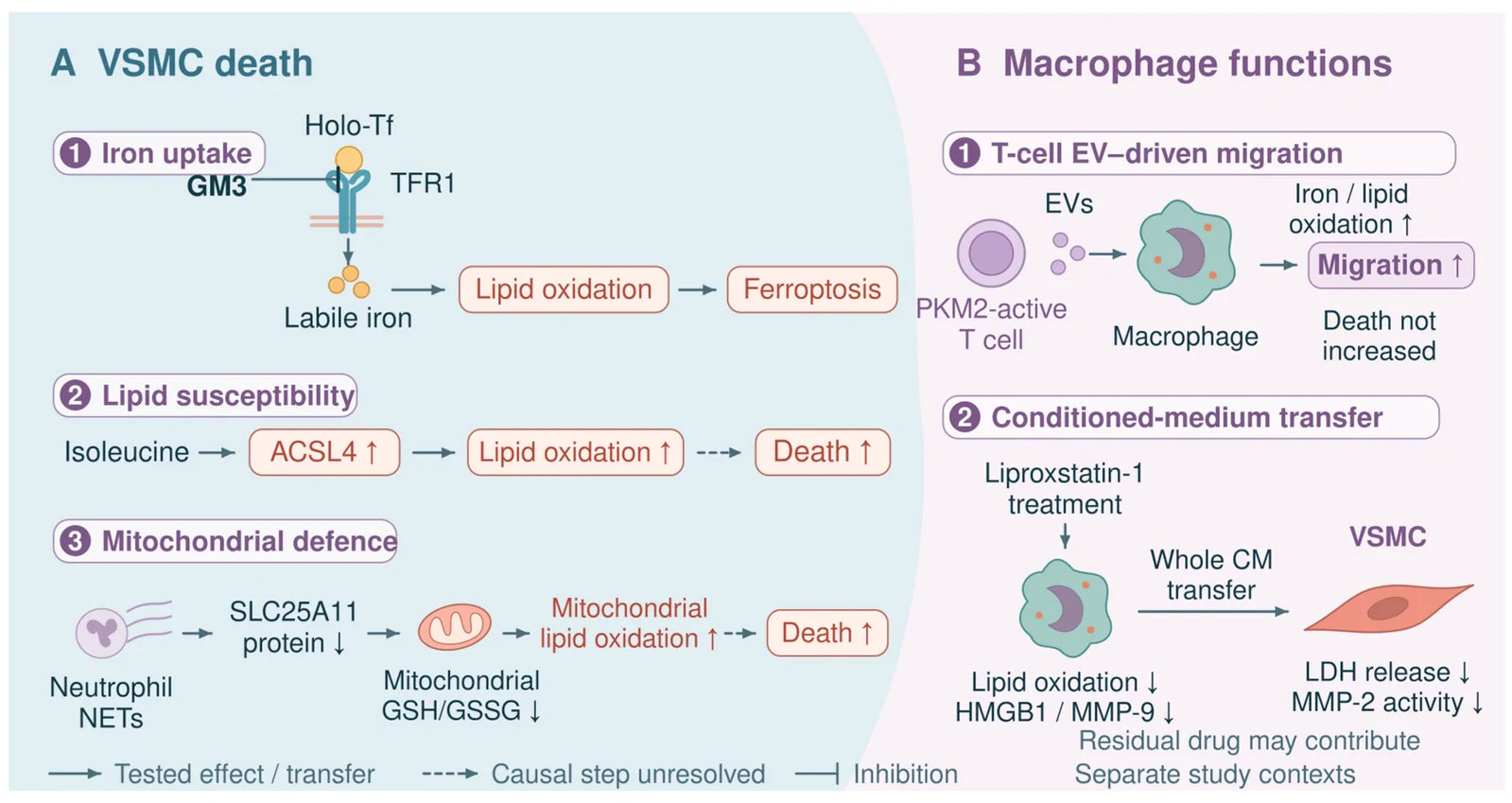
Distinct cellular consequences of redox perturbation in abdominal aortic aneurysm studies. (**A**) Cultured human vascular smooth muscle cell (VSMC) studies organised around (A1) iron uptake, (A2) lipid susceptibility and (A3) mitochondrial glutathione defence. (**B**) Macrophage contexts distinguishing (B1) migration without appreciably increased death from (B2) secretory output and conditioned-medium (CM) effects on recipient VSMCs. Panels represent separate studies, not a jointly validated network. Solid arrows indicate tested effects or transfer; blunt ends indicate inhibition; dashed terminal arrows identify incompletely resolved links between lipid oxidation and death. CM arrows represent whole-medium transfer, without assigning effects to individual mediators or excluding residual drug. Upward/downward arrows indicate changes relative to the experimental comparison. Blue-grey and grey-lilac backgrounds distinguish VSMC and macrophage contexts; gold circles denote iron. ACSL4, acyl-CoA synthetase long-chain family member 4; EV, extracellular vesicle; GM3, ganglioside GM3; GSH/GSSG, reduced/oxidised glutathione; HMGB1, high-mobility group box 1; holo-Tf, holo-transferrin; LDH, lactate dehydrogenase; MMP, matrix metalloproteinase; NET, neutrophil extracellular trap; PKM2, pyruvate kinase M2; SLC25A11, mitochondrial 2-oxoglutarate/malate carrier; TFR1, transferrin receptor 1.

Limiting VSMC death may preserve capacity for medial maintenance and repair [38,53,54,75,76,77]. Reducing macrophage recruitment or damaging output could support those surviving cells by easing inflammatory and proteolytic pressure on the surrounding matrix (Figure 2) [49,72,73,74,78]. These routes to wall protection may therefore reinforce one another. Preventing macrophage death without reducing injurious activity could instead sustain wall injury, making survival alone insufficient to predict the net effect on AAA. Current interventions do not separate the contributions of survival, recruitment and secretion to aortic outcome.

Neutrophils supply proteolytic and NET-associated inputs to matrix and VSMC injury [22,53,54], while endothelial BH4/eNOS regulation concerns oxidant production rather than a demonstrated death mechanism [58]. These findings identify potential sources of stress on neighbouring wall cells. Whether ferroptotic loss of either population contributes to AAA remains unresolved.

Blood product handling introduces a further distinction among macrophages. Haemoglobin-exposed CD163-high cells increase haemoglobin uptake [23], but whether preserving these cells protects the aorta remains unresolved. Uptake of soluble haemoglobin differs from erythrophagocytosis; the studies reviewed here establish neither ferroptosis susceptibility nor the aortic consequences of survival or loss in erythrophagocytic macrophage subsets. A comparable gap concerns adventitial fibroblasts: the redox exposures that could provoke ferroptotic injury and whether such injury impairs matrix maintenance or alters AAA growth have not been defined in these studies. The value of preserving an exposed cell population may therefore depend on whether its surviving cells sustain repair, dispose of blood products or continue to injure the wall (Table 1).

## 4. Experimental Therapeutic Opportunities and Translational Priorities

Therapeutic development requires redox processes that remain modifiable as AAA enlarges and interventions that reach the relevant wall compartment. Direct approaches alter iron handling or lipid peroxide accumulation, whereas upstream modulation changes inflammatory or metabolic inputs. Delivery systems can determine cellular exposure and enable expression of an antioxidant cargo.

Direct molecular and redox approaches include GM3/TFR1-dependent iron uptake and PCSK9-associated ferritin turnover. GM3/TFR1 experiments identify a cellular uptake mechanism, while Cadd4 reduces PCSK9 in VSMCs and aortic α-smooth muscle actin (α-SMA)-positive regions [38,41]. These findings connect intervention to target modulation, although the cellular selectivity of systemic dosing remains unresolved. For agents reaching both VSMCs and macrophages, structural cell survival and inflammatory cell recruitment and secretion should be assessed alongside matrix integrity and aortic growth. Relating these responses to target engagement would help determine whether the cellular effects reinforce or offset one another. Iron supports erythropoiesis and cellular metabolism, oxidised phospholipids participate in signalling and coagulation, and antioxidant pathways support immune and reparative functions. The bleeding phenotype in a separate acute systemic procoagulant lipid experiment illustrates the need to assess aortic benefit alongside the affected systemic function [45].

Upstream modulation changes the inputs that sustain wall cell stress. Suppressing NETs or EV release modifies inflammatory exposure, whereas dietary isoleucine restriction changes a metabolic input to lipid susceptibility [48,49,53]. RvD1 acts through FPR2 to limit macrophage redox and secretory responses [72]. GlycoRNA-rich membrane-coated nanoparticles couple reduced neutrophil accumulation with small interfering RNA (siRNA) against metallothionein 1 (MT1), adding delivery to this upstream strategy [79]. Systemic nutrient restriction and sustained NET suppression can also affect physiological functions beyond the aneurysm wall.

Delivery can provide local antioxidant support or introduce intracellularly expressed cargo, as illustrated by ROS-responsive selenium particles [67] and vesicles carrying mRNA encoding PEBP1 [69]. Cyclic arginine–glycine–aspartate (cRGD)-modified tea polyphenol nanoparticles combined aortic accumulation and ROS-triggered doxycycline release with carrier antioxidant activity; their greater reduction in dilatation than free drug or carrier alone supports evaluation of the formulation as a whole [80]. Oral doxycycline did not reduce two-year growth of small infrarenal AAA in a randomised trial [81]. Whether this delivery approach improves efficacy in established AAA requires direct testing.

Delivery also permits antioxidant enzyme augmentation and treatment response monitoring. Hyaluronic acid-coated peptide nanoparticles increased aortic expression of superoxide dismutase 2 (SOD2) from delivered mRNA and reduced enlargement in elastase models [82]. An oxidation-sensitive dextran formulation containing ultrasmall zinc-doped Prussian blue nanozymes (OZn) coupled antioxidant treatment to a urinary catalytic activity signal associated with treatment response in CaCl_2_-treated rats [83]. Interpretation of this signal requires accounting for cargo release and renal clearance as well as disease activity.

Delivery must also be assessed against systemic effects. The cRGD formulation reduced the hepatic and renal injury observed with equivalent-dose free doxycycline in mice [80], while MT1-siRNA and OZn formulations showed short-term tolerability at the tested doses [79,83]. Tea polyphenol carbon dots also lowered blood pressure [68], a systemic change that may contribute to aortic outcomes. Aortic and organ fluorescence profiles provide preliminary distribution and persistence information [80,83]; quantifying active cargo in wall cells and characterising its pharmacokinetics would clarify the exposure underlying these effects. Repeated-dose toxicity remains relevant to prolonged treatment.

Disease stage also determines the therapeutic question addressed by an experiment. Liproxstatin-1 reduced enlargement when given on days 14–27 in an elastase–BAPN protocol designed to treat pre-formed AAA [74], and selenium nanoparticles were administered four weeks after CaCl_2_ application [67]. Both schedules extend beyond initiation, but individual pretreatment confirmation of the lesion was not reported. Although delivery of mRNA encoding SOD2 was evaluated for its effects on both enlargement and rupture, dosing began on days 3 or 5 after induction [82]. To define efficacy against continued enlargement, these approaches need to be tested after imaging has confirmed baseline dilatation, with growth followed serially.

The lesion that supplies the redox stress also matters, because AAA models reproduce different components of human disease [84,85]. Ang II-infused *Apoe*-deficient mice develop branch-associated medial tears and intramural haematomas within days [86]. Blood trapped within a disrupted wall differs anatomically from an endoluminal ILT overlying chronically remodelled human AAA. Porcine pancreatic elastase protocols impose matrix injury, whereas periarterial CaCl_2_ initiates elastic network disruption and inflammation [87,88]. Wall iron deposits were also detected in a CaCl_2_ model reported to lack thrombus and vascular haemorrhage [26], showing that iron staining alone does not identify thrombus breakdown as the source of the iron. A shared increase in diameter or lipid oxidation markers therefore does not establish equivalent sources, durations or compartments of redox stress.

Periadventitial elastase combined with BAPN offers a longer window: persistent infrarenal enlargement and an ILT have been reported in combined protocols [87,89], with spontaneous rupture also reported in the chronic model [89]. These features permit investigation of growth after formation, but continued expansion can depend on lysyl oxidase inhibition; withdrawing BAPN stabilised an existing aneurysm and adding it restarted growth [87]. Protection must therefore be interpreted against the model’s constraints on matrix repair, remaining wall architecture and thrombus phenotype. Rupture of an acutely tearing Ang II aorta and failure of a chronically expanding wall also represent different therapeutic challenges. Adequately powered, model-appropriate rupture assessment, together with cell-resolved iron and lipid measurements, would help determine whether protection persists as wall architecture and redox exposure change.

Human validation must also account for disease stage and patient heterogeneity. Surgical specimens mainly represent advanced lesions; regional signals cannot be assumed to describe earlier growth [19,20]. Nevertheless, human data already identify variation relevant to redox biology. Active smoking was associated with oxidative stress and inflammatory transcriptional programmes, while preoperative growth correlated with different medial immune and adventitial lipid metabolism programmes [19]. Direct tissue measurements also associated smoking and hypercholesterolaemia with greater superoxide production [90]. Together with the regional effects of an ILT [17,18], these findings argue against a uniform exposure across patients or wall samples. They locate potential modifiers, but do not identify ferroptotic death or establish that a particular pathway explains faster growth.

Metabolic disease and medication use may also shape antioxidant responses. Cultured aortic VSMCs derived from diabetic AAA patients showed greater abundance of pentose phosphate pathway and antioxidant response proteins, but all five diabetic donors in this comparison were taking metformin, preventing separation of disease and drug effects [91]. Plasma hepcidin was inversely associated with aneurysm growth [44], while circulating NET markers were associated with rupture and study-defined rapid growth [54]. In the GM3 case–control study, GM3 showed a weak positive association with HDL cholesterol, and the groups differed in sex composition and HDL. Exploratory comparisons did not detect GM3 differences by sex or smoking status [38] but were insufficient to exclude subgroup effects. Linking these patient characteristics and regional ILTs to serial imaging and redox and survival measurements in defined wall cells would help distinguish changes accompanying enlargement from differences attributable to patient composition or lesion stage.

Table 2 compares experimental redox interventions and delivery strategies, including treatment timing and constraints on clinical translation.

## 5. Conclusions

Studies of human AAA tissue and experimental models implicate altered iron handling, phospholipid peroxidation and antioxidant defence in injury across thrombotic, medial and inflammatory regions of the wall. Experimental findings distinguish ferroptosis in cultured VSMCs from redox-dependent changes in coagulation, macrophage migration and secretion. The contrast between injurious iron uptake and protective iron retention further shows why the affected cellular function matters when choosing an intervention. Translation requires testing after confirmation of aneurysm formation, serial imaging and spatially matched human tissue. These approaches can test whether the relevant redox processes persist during enlargement, verify target engagement in the intended wall cells and determine how local protection relates to systemic effects.

## Figures and Tables

**Table 1:** Ferroptosis and other redox mechanisms linking cellular functions to AAA outcomes.

Study and Process	Experimental Context and Timing	Redox Mechanism and Cellular Consequence	Aortic Outcome
Ferroptosis demonstrated in cultured VSMCs			
GM3/TFR1 [38]	Human VSMCs; Ang II mice. Pre-induction genetics; early GM3.	*ST3GAL5* loss increases TFR1-dependent iron uptake, lipid peroxidation and VSMC death. Rescue of death by both Fer-1 and *TFRC* knockdown identifies iron uptake as a driver of ferroptosis in this cellular setting.	SMC *St3gal5* loss worsened AAA; systemic GM3 reduced formation.
Ferroptosis supported in cultured VSMCs			
Isoleucine/ACSL4 [48]	Human VSMCs; Ang II mice. Pre-induction bacterial/dietary exposure.	Isoleucine increases ACSL4-associated lipid oxidation and VSMC death. Fer-1 rescues death, whereas *ACSL4* knockdown reduces oxidation; these findings support ferroptosis, with the iron/ACSL4-to-death link incompletely resolved.	Bacterial *ilvE* loss and isoleucine restriction reduced AAA.
SLC25A11/mitochondrial GSH [54]	NET-exposed human SMCs; Ang II mice. Pre-induction drugs/genetics.	NETs compromise SLC25A11-related mitochondrial GSH defence, increasing lipid oxidation and death. SLC25A11 augmentation rescues PI-positive death; Fer-1 rescue is assessed using CCK-8, supporting ferroptosis while leaving death-specific drug rescue unresolved.	NET inhibition/Fer-1 reduced AAA; SLC25A11 rescue was tested in culture.
Redox-associated mechanisms of cellular defence and inflammatory stress			
GPX4 defence [52]	MOVAS; elastase–BAPN/Ang II mice. Pre-induction global overexpression.	GPX4 augmentation reduces aortic lipid oxidation and suppresses IL-6/JAK1/STAT3 signalling in MOVAS, supporting antioxidant and anti-inflammatory functions. These experiments do not establish a VSMC death endpoint with functional evidence identifying ferroptosis as its mechanism.	Global overexpression reduced AAA formation and inflammation.
PCSK9/ferritin turnover [41]	Mouse VSMCs; PPE/Ang II mice. Pre-induction genetics; early Cadd4.	PCSK9-linked lysosomal ferritin turnover alters cellular iron, lipid peroxidation and metabolic viability. Rescue by lysosomal inhibition supports this oxidative injury route, without isolating NCOA4 dependence.	Cadd4 reduced dilatation and matrix injury.
Antioxidant responses [50,51,59,63]	VSMCs; Ang II [50,51,63]/PPE [59] mice. Early [51,63]; onset unclear [50,59].	Fer-1 suppresses lipid oxidation and preserves metabolic viability [50,51], with *Slc7a11*/*Gpx4* involvement tested by silencing [50]. Menaquinone-4 protection involves NRF2/GCLM-linked glutathione homeostasis [59], while PRDX2 augmentation limits peroxide stress [63].	Aortic injury or diameter decreased [50,51,59,63]; elastin/collagen changes were not significant in [59].
NET reduction [53]	NET-exposed human SMCs; Ang II mice. Pre-induction drugs; early MSC-EVs.	NET exposure depletes GSH and increases iron and oxidative stress in human SMCs. MSC-EVs reduce NET release upstream, whereas Fer-1/PI3K interventions improve the SMC redox and metabolic viability response.	NET reduction attenuated AAA.
SENP3/CTH/H_2_S [73]	Mouse macrophages; Ang II/CaCl_2_ mice. Pre-induction genetics; early drugs.	SENP3 de-SUMOylates CTH and promotes its degradation, lowering H_2_S availability. SENP3 deficiency preserves CTH-dependent redox support, limiting iron accumulation, oxidative injury and pro-inflammatory transcription; CTH perturbation weakens this protection.	Myeloid *Senp3* loss protected; CTH inhibition weakened this benefit.
Antioxidant and mRNA delivery [67,68,69]	Cultured VSMCs; CaCl_2_ [67,69]/Ang II [68] mice. Delayed [67]; pre-induction [68]; onset unclear [69].	Selenium delivery increases antioxidant defence and metabolic viability [67]. Tea polyphenol carbon dots reduce oxidative injury and apoptosis [68]. EVs carrying mRNA encoding PEBP1 reduce ROS and apoptosis in cultured mouse VSMCs [69].	Dilatation decreased [67,68,69]; blood pressure also decreased in [68].
Redox-associated functions in wall maintenance and inflammatory or thrombotic injury			
Hepcidin/iron retention [44]	SMCs; Ang II mice. Pre-induction genetics; early anti-LCN2.	Hepcidin-dependent iron retention supports SMC autophagy. Hepcidin loss increases LCN2 and impairs autophagy; LCN2 neutralisation restores it, identifying a protective iron-handling function.	SMC hepcidin loss worsened AAA; LCN2 neutralisation attenuated injury.
T-cell EVs [49]	Macrophages; elastase mice. Pre-induction genetics/EV inhibition.	T-cell EVs increase macrophage iron, lipid oxidation and migration without appreciably increasing death. Iron chelation, Fer-1 and liproxstatin-1 suppress migration, linking iron/lipid oxidation to the recruitment of surviving cells.	T-cell PKM2 loss/EV inhibition reduced macrophage accumulation and AAA.
Oxidised phospholipids [45]	Human wall/ILT observations; Ang II mice. Pre-induction genetics; early lipids/factor Xa inhibition.	Oxidised phospholipids increase coagulation. Mouse lipid enzyme and factor Xa interventions implicate thrombotic function in AAA, complementing detection of oxidised lipids in human wall/ILT.	Deletion or systemic lipid dosing reduced AAA; a separate acute dosing test caused bleeding.
Macrophage–VSMC interaction [72,74]	Macrophages/recipient VSMCs; elastase ± BAPN mice. Early [72,74]; delayed [74].	RvD1/FPR2 limits NOX2-associated oxidation and HMGB1 release [72]; liproxstatin-1 reduces lipid oxidation and HMGB1/MMP-9 output [74]. Medium from treated macrophages induces less recipient-VSMC MMP-2 activity [72,74] and LDH release [74]; residual liproxstatin-1 may also contribute to the recipient-cell response.	Early RvD1 [72] and early/delayed liproxstatin-1 [74] reduced enlargement.

Ferroptosis attribution. Review-specific criteria are informed by [5,37]. Demonstrated: measured death and lipid oxidation, with death rescue identifying dependence on both iron availability and lipid peroxidation. Supported: measured death with lipid oxidation, but incomplete identification of the lethal mechanism. Redox-associated: ferroptotic death is not established; includes causally supported redox functions. Categories concern cellular ferroptosis attribution, not study quality. CCK-8 measures metabolic viability. Lipid pathway perturbation alone does not establish iron dependence; aortic benefit does not establish mediation by ferroptosis. Timing. Pre-induction precedes induction; early starts at or shortly after induction. Delayed does not imply pretreatment lesion confirmation; unclear indicates unspecified onset. Constitutive genetics test susceptibility. References distinguish studies within grouped rows. Table 1 abbreviations. AAA, abdominal aortic aneurysm; ACSL4, acyl-CoA synthetase long-chain family member 4; Ang II, angiotensin II; BAPN, β-aminopropionitrile; CCK-8, Cell Counting Kit-8; CTH, cystathionine γ-lyase; EV, extracellular vesicle; Fer-1, ferrostatin-1; FPR2, formyl peptide receptor 2; GCLM, glutamate-cysteine ligase modifier subunit; GM3, ganglioside GM3; GPX4, glutathione peroxidase 4; GSH, glutathione; HMGB1, high-mobility group box 1; IL-6, interleukin-6; ILT, intraluminal thrombus; JAK1, Janus kinase 1; LCN2, lipocalin 2; LDH, lactate dehydrogenase; MMP, matrix metalloproteinase; MOVAS, mouse aortic vascular smooth muscle cell line; MSC, mesenchymal stromal cell; NCOA4, nuclear receptor coactivator 4; NET, neutrophil extracellular trap; NOX2, NADPH oxidase 2; NRF2, nuclear factor erythroid 2-related factor 2; PCSK9, proprotein convertase subtilisin/kexin type 9; PEBP1, phosphatidylethanolamine-binding protein 1; PI, propidium iodide; PI3K, phosphoinositide 3-kinase; PKM2, pyruvate kinase M2; PPE, porcine pancreatic elastase; PRDX2, peroxiredoxin 2; ROS, reactive oxygen species; RvD1, resolvin D1; SENP3, SUMO-specific peptidase 3; SLC7A11, solute carrier family 7 member 11; SLC25A11, mitochondrial 2-oxoglutarate/malate carrier; SMC, smooth muscle cell; STAT3, signal transducer and activator of transcription 3; SUMO, small ubiquitin-like modifier; TFR1, transferrin receptor 1; VSMC, vascular smooth muscle cell.

**Table 2:** Experimental redox interventions and delivery strategies for AAA.

Strategy and Study	Cellular Focus and Model	Treatment Window	Therapeutic Advance and Supporting Evidence	Key Translational Constraint
Direct molecular and redox interventions				
GM3/TFR1 Zhang 2024 [38]	VSMCs; Ang II mice	Early: day 0 onward, every 3 days.	A pharmacological route to limit iron uptake: GM3 bound TFR1 and reduced uptake in cultured VSMCs; systemic dosing reduced AAA formation.	Formation-stage efficacy; long-term effects of systemic iron uptake modulation remain unresolved.
PCSK9 degradation Cadd4 Xia 2026 [41]	VSMCs; PPE and Ang II mice	Early: day 3 onward, every other day.	PCSK9 decreased in aortic α-SMA-positive regions; PCSK9 degradation in cells was proteasome-dependent. Dilatation and matrix injury also decreased.	Systemic peptide bioavailability, cell selectivity and long-term safety need characterisation.
Lipid radical trapping Liproxstatin-1 Krebs 2025 [74]	Macrophage–SMC interaction; elastase ± BAPN mice	Early: days 0–7. Delayed: days 14–27 with BAPN.	Reduced enlargement with delayed dosing extends lipid radical trapping beyond induction. Lower tissue lipid oxidation provides a pharmacodynamic readout.	Pretreatment lesion confirmation was not reported; rupture events were insufficient to establish protection against rupture.
Upstream modulation of inflammatory exposure				
RvD1/FPR2 Filiberto 2022 [72]	Macrophages; elastase mice	Early: days 1–13.	FPR2-dependent protection connects modulation of macrophage NOX2-associated oxidation and secretory activity with reduced aortic dilatation.	Global receptor deletion does not isolate macrophage action; treatment of established lesions remains untested.
MT1 siRNA delivery GlycoRNA-NP-siMT1 Zhang 2025 [79]	Neutrophil/NET input; PPE mice	Early: days 1, 3 and 7 after PPE.	Combines reduced neutrophil accumulation with siRNA delivery: aortic MT1 and NET burden fell, with reduced dilatation. Carrier and control-RNA comparisons address both components.	Early treatment; in vivo cell-selective silencing and the consequences of sustained NET suppression remain unresolved.
Delivery of antioxidant activity and monitoring of treatment response				
ROS-responsive selenium delivery He 2025 [67]	VSMCs; CaCl_2_ mice	Delayed: dosing four weeks after induction.	Aortic accumulation supports lesion delivery, while antioxidant proteins increased in cultured VSMCs. Delayed dosing reduced dilatation.	Pretreatment lesion confirmation was not reported. Pharmacokinetics and long-term selenium exposure need evaluation.
mRNA encoding SOD2 HA-coated peptide NPs Yan 2025 [82]	Macrophage uptake; elastase perfusion or elastase + TGF-β blockade mice	Early: days 5, 8, 11 after perfusion; days 3, 5, 7, 9 with TGF-β blockade.	mRNA delivery increased aortic SOD2 and reduced oxidative injury. Reduced enlargement and improved survival in the rupture model support antioxidant enzyme augmentation.	Early dosing without individual baseline confirmation; TGF-β blockade limits extrapolation of rupture protection.
cRGD-modified tea polyphenol NPs/doxycycline Xu 2025 [80]	VSMCs and macrophages; Ang II mice	During induction: every 2 days throughout modelling.	Targeting increased aortic accumulation. Compared with free drug or carrier alone, loaded targeted particles reduced diameter more; versus equivalent-dose free doxycycline, they also reduced hepatic and renal injury in the tested mouse regimen.	Multiple drug/carrier actions; formation-stage efficacy. Oral doxycycline did not slow AAA growth in the clinical trial [81]; this formulation remains untested in established AAA.
OZn antioxidant nanozyme with urinary readout Liu 2025 [83]	VSMCs and inflammatory cells; CaCl_2_ rats	Early: day 2, then twice weekly for 3 weeks. Delayed: day 10, every 3 days for four doses.	ROS-responsive antioxidant delivery reduced aortic injury. Urinary nanozyme catalytic activity supplied an experimental readout associated with treatment response.	The urinary readout depends on release and renal clearance; its disease specificity and clinical validity are unestablished.

Interpretation. Cellular focus does not imply exclusive targeting in vivo. Tissue accumulation, intracellular cargo expression, target modulation and downstream biochemical responses represent distinct forms of evidence. Antioxidant activity or aortic benefit alone does not establish mediation by ferroptosis. Timing. Day 0 denotes induction. Early treatment starts during lesion development; delayed treatment starts later in the protocol but does not imply individual pretreatment confirmation of AAA. OZn response monitoring used a separate regimen beginning on day 10, with dosing every 2 days. The GlycoRNA-NP-siMT1 row reports the main PPE treatment schedule. Abbreviations. AAA, abdominal aortic aneurysm; Ang II, angiotensin II; BAPN, β-aminopropionitrile; cRGD, cyclic arginine–glycine–aspartate peptide; FPR2, formyl peptide receptor 2; GM3, ganglioside GM3; HA, hyaluronic acid; MT1, metallothionein 1; NET, neutrophil extracellular trap; NOX2, NADPH oxidase 2; NP, nanoparticle; OZn, oxidation-sensitive dextran containing ultrasmall zinc-doped Prussian blue; PCSK9, proprotein convertase subtilisin/kexin type 9; PPE, porcine pancreatic elastase; ROS, reactive oxygen species; RvD1, resolvin D1; siRNA, small interfering RNA; SMC, smooth muscle cell; SOD2, superoxide dismutase 2; TFR1, transferrin receptor 1; TGF-β, transforming growth factor β; VSMC, vascular smooth muscle cell; α-SMA, α-smooth muscle actin.

## Data Availability

No new data were created or analysed in this study. Data sharing is not applicable to this article.
